# Clinical phenotype and functional influence of *GRIN2A* variants in epilepsy‐aphasia syndrome

**DOI:** 10.1002/epi4.13057

**Published:** 2024-10-30

**Authors:** Lu Zhang, Yiran Duan, Rui Ma, Jiaqi Han, Na Pan, Lehong Gao, Yuping Wang

**Affiliations:** ^1^ Department of Neurology Xuanwu Hospital, Capital Medical University Beijing China; ^2^ Center of Epilepsy, Beijing Institute for Brain Disorders Capital Medical University, Ministry of Science and Technology Beijing China; ^3^ Beijing Institute of Brain Disorders, Collaborative Innovation Center for Brain Disorders Capital Medical University Beijing China; ^4^ Present address: Department of Neurology Beijing Friendship Hospital, Capital Medical University Beijing China; ^5^ Present address: Department of Endocrinology, Genetics and Metabolism National Center for Children's Health, Beijing Children's Hospital, Capital Medical University Beijing China

**Keywords:** child‐hood epilepsy, electrophysiological studies, genetics, N‐methyl‐D‐aspartate receptors

## Abstract

**Objective:**

N‐methyl‐D‐aspartate receptors are glutamate‐gated ion channels that play a crucial role in brain function. Numerous inherited or de novo variants in the *GRIN2A* gene, encoding the GluN2A subunit of the receptor, have been identified in patients with epilepsy. In addition, it is worth noting that *GRIN2A* variants exhibit a strong correlation with epilepsy‐aphasia syndromes, a group of age‐dependent epileptic, cognitive, and language disorders with a characteristic electroencephalographic pattern.

**Methods:**

Whole exome sequencing was conducted in enrolled patients with epilepsy‐aphasia syndromes, and *GRIN2A* variants were screened. The conservation of substituted residues, conformational changes of mutant subunits, and in silico predictions of pathogenicity were thoroughly assessed in our study. Functional alterations of the variants were examined using whole‐cell voltage‐clamp current recordings while the relative surface expression levels of subunit proteins were assessed via immunofluorescence assays. A summary of previously published *GRIN2A* missense variants was conducted to investigate the genotypic‐phenotypic‐functional correlations.

**Results:**

Two missense *GRIN2A* variants (c. 2482A >G/p. M828V, c. 2627 T >C/p. I876T) were identified, which are located in the transmembrane helix M4 and C‐terminus domain of the GluN2A subunit, respectively. Both variants exhibited reduced current density of NMDARs and surface/total expression levels of GluN2A subunits, while M828V showed a decreased extent of desensitization as well. A further summary of the previously reported *GRIN2A* variants demonstrated that more variable phenotypes were observed for variants situated in the C‐terminus domain or those with loss‐of‐function effects.

**Significance:**

Our study expands the phenotypic and functional range of *GRIN2A*‐related disorders. In order to optimally establish the domain‐function‐phenotype correlations in *GRIN2A* variants, it is imperative to gather a more extensive set of clinical and functional data.

**Plain Language Summary:**

This study has identified two genetic variants of the *GRIN2A* gene in patients with epilepsy‐aphasia syndrome. We assess the variants' harmfulness through a variety of functional experiments, including evaluating the expression level of the mutated protein and the resulting changes in electrophysiological activities. Also, we reviewed previously published papers about *GRIN2A* variants in epilepsy to learn more about the correlations between their locations, functional changes, and clinical manifestations.

**Key Points**

- Two *GRIN2A* variants in the transmembrane M4 and C‐terminus domains were first functionally confirmed to be epilepsy‐aphasia syndrome related.
- Variants located in the lower M4 segment of the GluN2A subunit may cause loss‐of‐function of NMDA receptors through reduced receptor biogenesis.
- *GRIN2A* missense variants located in the C‐terminus domain or those with loss‐of‐function effects demonstrate more variable phenotypes.

## 1 INTRODUCTION

N‐methyl‐d‐aspartate receptors (NMDARs) are ligand‐gated ionotropic channels that mediate excitatory synaptic transmission and are critical for brain development, synaptic plasticity, and other higher brain functions such as learning and memory. Most conventional NMDARs contain two GluN1 subunits that bind glycine (Gly) and two GluN2A‐D subunits that bind glutamate (Glu).^1^ The membrane structure of each subunit is made up of four parts: the extracellular amino‐terminal domain (ATD), which binds allosteric modulators; the ligand‐binding domain (LBD), which has segments S1 and S2 that mediate agonist binding; the transmembrane domain (TMD), which has segments M1–M4 that make up the channel pore; and the intracellular C‐terminus domain (CTD), which facilitates receptor trafficking. Activated NMDARs primarily mediate the calcium influx.^2^

Genetic and functional dysregulation of NMDARs can lead to neurological and psychiatric disorders. The expeditious development and widespread availability of sequencing techniques have facilitated the genetic diagnosis of individuals with overlapping phenotypes. An illustration of this can be the identification of variants in *GRIN2A*, the gene that encodes GluN2A subunits in NMDARs, being the predominant monogenic cause of epilepsy‐aphasia spectrum (EAS) syndrome.^3^, ^4^, ^5^ EAS is a heterogeneous group of age‐dependent childhood‐onset disorders characterized by sleep‐activated centrotemporal discharges, focal seizures, language skill impairments to varying degrees, and rare cognitive deficits. Self‐limited epilepsy with centrotemporal spikes (SeLECTS) is on the mild end of the spectrum, while early‐onset epileptic encephalopathy (EOEE) is on the severe end. Other phenotypes range from epileptic encephalopathy with spike‐and‐wave activation in sleep (EE‐SWAS) to Landau–Kleffner syndrome (LKS).

Clinical phenotype heterogeneity, including some interfamilial heterogeneity, can be observed in patients with variants. The functional consequences of variants, influenced by their type and location, can partially contribute to the heterogeneity in clinical phenotypes.^6^, ^7^, ^8^, ^9^, ^10^, ^11^ However, time and resource constraints have prevented functional investigation of most disease‐related variants. The lack of understanding in this area hinders the identification of pathogenic variants and the characterization of the phenotypic spectrum, widening the gap between the abundant yet complex genetic information and clinical management.

In this study, trio‐based whole‐exome sequencing in patients with EAS identified two *GRIN2A* missense variants. We summarized the patients' clinical features and assessed the functional modifications of these variants in vitro. Also, we reviewed previously published papers about missense *GRIN2A* variants in epilepsy to learn more about the correlations between subregional effects, functional changes, and phenotypic diversity.

## 2 METHODS

### 2.1 Participants and sequencing

Patients diagnosed with EAS were enrolled in outpatient units at the Neurology and Pediatric Department of Xuanwu Hospital from May 2017 to December 2020, inclusion criteria and exclusion criteria were as previously described.^12^ Patients' and their parents' genomic DNA (if available) were extracted from peripheral blood samples, and the whole‐exome sequencing was conducted by BGI Genomics. Raw data that passed the quality filter was aligned to Genome Reference Consortium Human Build 37 (GRCh37). *GRIN2A* variants were annotated based on the transcript NM_000833.4 to collect amino acid alterations. Potential pathogenic variants were validated by Sanger sequencing.

This study was approved by the Ethics Committee of Xuanwu Hospital Capital Medical University. Informed consent was obtained from all individual participants' legal guardians.

### 2.2 Evaluation of pathogenicity by in silico methods

Ten prediction tools (Table S1) were used to evaluate the pathogenicity of each variant. The PhastCons100way score was used to quantify the evolutionary conservation of residues with detected changes, and a score of 1 indicates the greatest conservation.^13^ The allele frequencies of identified variants in populations were obtained from the Genome Aggregation Database (gnomAD). Protein conformations of the wild‐type (WT) and mutant subunits were modeled by the Iterative Threading ASSEmbly Refinement (I‐TASSER) software,^14^, ^15^ with three‐dimensional structures, polar contacts, and atoms involved shown in PyMOL 2.5.5 (Schrödinger).

### 2.3 cDNA construction, cell culture, transfection

Human *GRIN1* cDNA (NM_007327) was cloned into the NheI / BamHI restrictive sites of the GV417 vector, coupled with a red fluorescent reporter protein, mCherry. While *GRIN2A*‐WT (NM_000833) and *GRIN2A*‐MUT (M828V, I876T) were cloned and inserted into the XhoI / KpnI restriction sites of the EGFP‐containing GV230 vector. Plain *GRIN1* plasmids without mCherry were constructed within GV712 vectors to avoid any visual disturbance to the fluorescent labels during surface expression testing of GluN2A subunits. All plasmids were constructed by Genechem, and sequencing was confirmed.

Human embryonic kidney (HEK) 293 cells were grown in Dulbecco's Modified Eagle's Medium (Gibco), supplemented with 10% fetal bovine serum (Gibco) and 10 U/mL penicillin/streptomycin (Gibco). *GRIN1*‐WT constructs were co‐transfected with either *GRIN2A*‐WT/MUT constructs using Lipofectamine 2000 (Invitrogen). To protect against NMDAR‐mediated toxicity, 0.2 mM D, L‐2‐amino‐5‐phosphonovaleric acid (Sigma), and 1 mM kynurenic acid (Sigma) were added to the culture medium.

### 2.4 Immunofluorescence assays of surface and total GluN2A subunits

Immunofluorescence assays were performed 24 hours after transfection of *GRIN1* and *GRIN2A‐EGFP* plasmids. Cells were sequentially incubated with a rabbit anti‐GFP antibody (1:1000; Abcam) and an anti‐rabbit antibody conjugated to Alexa Fluor 647 (1:1000; Proteintech). After that, the cells were fixed in 4% paraformaldehyde for 10 min and subsequently mounted on glass slides. Images were captured using a CSIM 100 confocal microscope (SUNNY). The intensity of surface and total expression of the GluN2A protein were measured using ImageJ2 (National Institutes of Health) and compared between the WT and mutant groups.

### 2.5 Whole‐cell recordings

Patch‐clamp experiments were performed 24–48 hours after transfection of *GRIN1‐*mcherry and *GRIN2A‐*EGFP plasmids. Cells were live‐imaged through fluorescence microscopy (Olympus), and those expressing both transfected subunits were used for recordings.

The recording chamber was perfused with the extracellular solution using the following recipe (in mM): 135 NaCl, 5 KCl, 2 CaCl_2_, and 10 HEPES at pH 7.4. Sucrose adjusted the osmolarity to 300 mOsm. Patch pipettes were made on the P‐1000 micropipette puller (Sutter). The internal solution filled in the pipettes was composed of (in mM) 140 CsCl, 2 MgATP, 10 EGTA, and 10 HEPES at pH 7.3, and the osmolarity was 300–310 mOsm.^9^ The whole‐cell current responses were acquired by Axon Multiclamp 700B amplifier (Molecular Devices) with pClamp 10.5 software (Molecular Devices) and digitized at 20 kHz using AXON Digidata 1500A digitizer (Molecular Devices). Micropipette positioning was controlled by MPC‐325 Multi‐Micromanipulator Systems (Sutter). HEK 293 cells were clamped at‐70 mV. A concentration of 1 mM Glu and 0.1 mM Gly, which proved to be maximally effective,^11^ was applied to the extracellular solutions for 2.5 s by a fast perfusion system (MappingLab) to activate NMDARs.

Recordings with a leak current that is less than 10% of the peak current is considered acceptable. Current measurements and analysis were performed with Clampfit 11 (Molecular Devices). The steady‐state current amplitude (Iss) was obtained at the end of the Glu/Gly application, while Ipeak stands for the peak current amplitude. Percent desensitization (% Desensitization) was calculated as 100 × (1‐Iss/Ipeak) %. Rise time, measured from the duration of 10% to 90% of the amplitude, indicates NMDAR activation kinetics. A two‐term exponential function was used to estimate the deactivation time course, with τ_fast_ and τ_slow_ representing the deactivation time constants for the fast and slow components, respectively.

### 2.6 Correlation analysis of *GRIN2A* variant domains, phenotypes, and functional consequences

We systematically searched PubMed (up to April 15, 2023) using the terms “*GRIN2A*” and “epilepsy” for reports and studies about potential pathogenic *GRIN2A* variants. This retrieval of functional, genetic, and clinical data exclusively included missense variants. We individually recorded the clinical information of identical variants in different individuals. Variants were functionally stratified as N/A (no data available), neutral (functions similar to WT), complex (paradoxical effects), GOF (gain‐of‐function), or LOF (loss‐of‐function). Clinical phenotypes were categorized into undefined focal seizures, benign epilepsy with centrotemporal spikes (BECTS), atypical benign partial epilepsy (ABPE), continuous spike–waves during slow‐wave sleep syndrome (CSWSS), LKS, EE & other ID/DD (epileptic encephalopathy & other intellectual/developmental delay) or other epilepsy types. We didn't utilize the updated version of terminology of epilepsy syndromes because most of the studies that we retrieved were using the older terms. *GRIN2A* variants from the ClinVar database were also retrieved (up to April 15, 2023) to assess potential bias between clinical scenarios and published studies.

### 2.7 Statistical analysis

All quantitative data values were expressed as mean ± SEM. Graphpad Prism8 was used for statistical analysis. One‐way ANOVA with Dunn's multiple comparison test was applied for immunofluorescence and electrophysiological data. *p* < 0.05 was considered statistically significant.

## 3 RESULTS

### 3.1 Identification of *GRIN2A* variants

**FIGURE 1:**
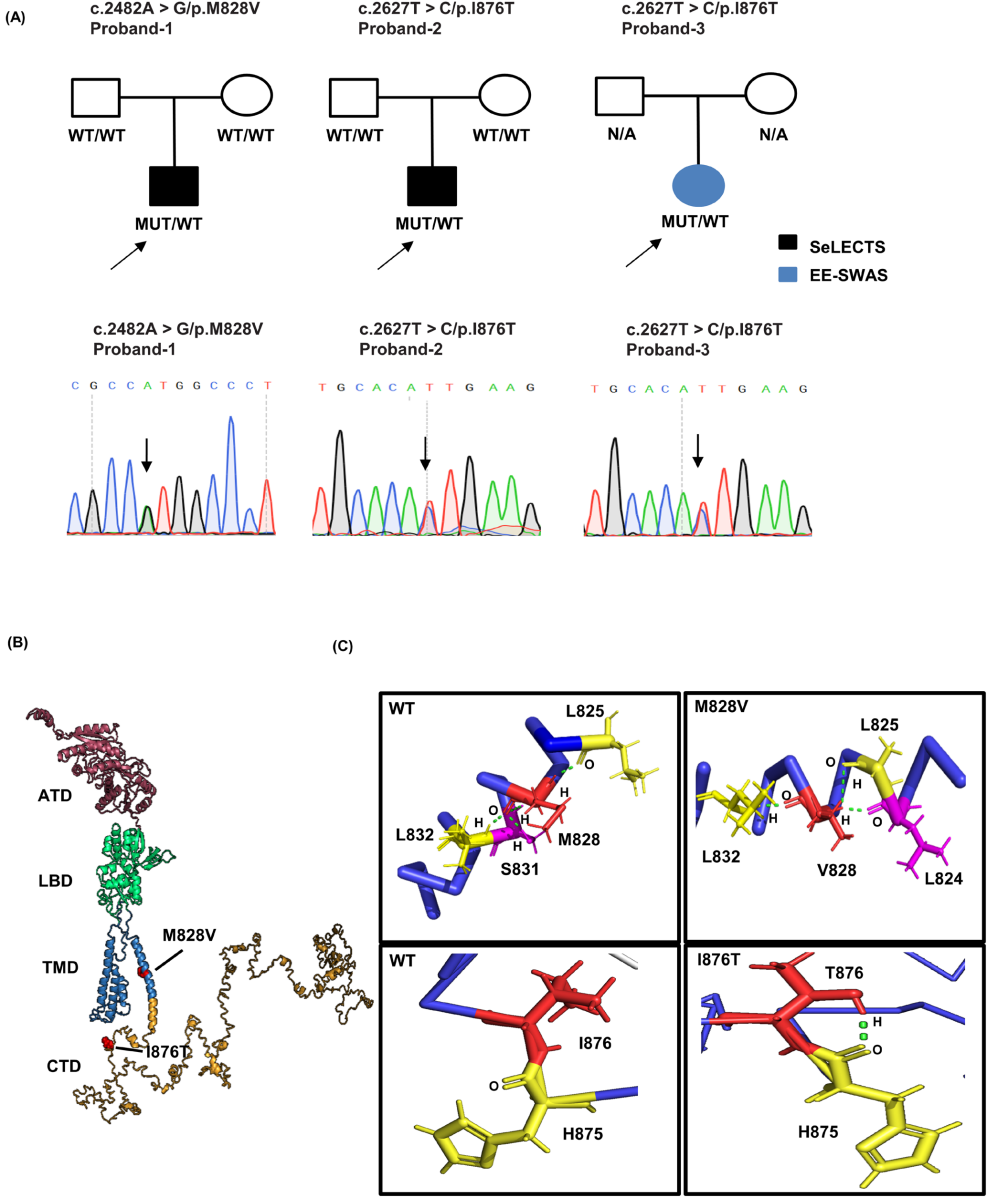
Genetic information about two identified *GRIN2A* variants from three probands and their locations in the three‐dimensional model of the GluN2A subunit. **(A)** Pedigrees and the Sanger sequencing traces of three cases with family information and their corresponding phenotypes. c.2627 T > C/p.I876T is a recurrent variant in proband‐2 and 3. Arrows indicate the probands and the variants' positions. Individuals with the *GRIN2A* variant are indicated by MUT/WT, and variant‐negative individuals are indicated by WT/WT. (B) Domain location of identified missense variants (M828V and I876T) in the three‐dimensional structure of the GluN2A subunit. Positions with affected residues are highlighted in red. (C) Hydrogen bonding connections with surrounding amino acids in the WT and mutant situations. Variant residues found in patients are colored red, and the magnet one was used to imply the altered connections before and after mutation. Atoms involved to form hydrogen bonds were indicated. WT, wildtype; MUT, mutant; SeLECTS, self‐limited epilepsy with centrotemporal spikes; EE‐SWAS, epileptic encephalopathy with spike‐and‐wave activation in sleep; ATD, amino‐terminal domain; LBD, ligand‐binding domain; TMD, transmembrane domain; CTD, carboxyl‐terminal domain. Amino acid: A, Alanine, aliphatic; H, Histidine, basic; I, Isoleucine, aliphatic; L, Leucine, aliphatic; M, Methionine, sulfur‐containing; T, Threonine, hydroxylic; V, Valine, aliphatic.

Two heterozygous missense *GRIN2A* variants were detected in three unrelated probands (Figure 1A). A de novo c. 2482A >G/p. M828V variant was observed in a SeLECTS patient. The variant c. 2627 T >C/p. I876T was recurrent in two individual cases, one of which in the SeLECTS patient was confirmed to be de novo through family segregation analysis, while the other proband with EE‐SWAS lacked any parental genetic information. Table 1 summarizes the cDNA alterations and amino acid changes, along with the clinical and genetic information of the probands.

**TABLE 1:** Summary of patients' information.

	Patient 1	Patient 2	Patient 3
cDNA change	c.2482A > G	c.2627 T > C	c.2627 T > C
Protein change	p. Met828Val	p. Ile876Thr	p. Ile876Thr
Protein domain	TMD M4	CTD	CTD
Inheritance	De novo	De novo	N/A
Family History	None	None	None
Gender	Male	Male	Female
Diagnoses	SeLECTS	SeLECTS	EE‐SWAS
Seizure Type	Focal seizures	Focal seizures	Focal seizures
Onset age (year)	5	9	6
Age at study (year)	12	15	23
ID/DD	–	–	–
Speech	–	Imprecise articulation (interictal)	
Sound in the throat (ictal)	Sound in the throat (ictal)		
EEG	Bilateral centrotemporal spikes	Bilateral centrotemporal spikes	Bilateral centrotemporal spikes, some multifocal parietal discharges
Spike–wave index	70%	50%–60%	80%–90%
MRI/CT	Normal	Normal	Normal
Dipole distribution in MEG	Left central sulcus	Left temporal lobe	Bilateral precentral gyrus
Medications	VPA	VPA LEV	VPA LEV steroids
Seizure outcome	3–5 times/year	Seizure‐free	Seizure‐free
Additional features	History of birth asphyxia	History of febrile seizures	ADHD, impaired memory

Abbreviations: ADHD, attention‐deficit/hyperactivity disorder; CTD, C‐terminal domain; EE‐SWAS, epileptic encephalopathy with spike‐and‐wave activation in sleep; ID/DD, intellectual delay/developmental delay; LEV, levetiracetam; N/A, nonapplicable; SeLECTS, self‐limited epilepsy with centrotemporal spikes; TMD, transmembrane domain; VPA, valproate.

The variant M828V is situated within the M4 segment of the TMD, while the I876T variant is located in the CTD (Figure 1B). We further examined their interactions with neighboring residues on the subunit's 3D structures modeled using I‐TASSER. (Figure 1C). Within the WT GluN2A subunit, residue M828 forms hydrogen bonds with residues L825, S831, and L832. Residue I876 failed to form any interactions. In contrast, the M828V variant exhibited new bonding with L824, but lost the connection with S831. The I876T formed a hydrogen bond with H875. These modifications suggest that the subunit may undergo potential structural alterations, which could impact its functional properties.

M828V and I876T were considered damaging by 6 and 3 of the 10 in silico prediction tools we employed. The PhastCons100way scores suggested that both variants are highly evolutionarily conserved, indicating their functionally prominent role in NMDARs (Table S1). The variant M828V was absent from the gnomAD database, while I876T was found across all populations and had an even higher frequency in East Asian populations (Table S2).

### 3.2 Clinical information for patients with *GRIN2A* variants

The M828V variant occurred in a 12‐year‐old boy with birth asphyxia, normal development, and no family history of epilepsy or febrile seizures. At five years old, he had his first seizure while sleeping. Focal seizures with right‐sided facial twitching and eye blinking, as well as salivating, were described. Video electroencephalogram (EEG) monitoring showed bilateral centrotemporal spikes and a 70% spike–wave index during non‐rapid eye movement (NREM) sleep. His brain MRI imaging was negative, while magnetoencephalography (MEG) revealed left central sulcus dipoles. Both clinical manifestations and diagnostic workups support the diagnosis of SeLECTS. With a daily dosage of Valproate at 1000 mg, the patient still experiences 3–5 nocturnal seizures per year. No speech problems or any developmental issues were presented in the interictal period.

Two probands with the I876T variant were from different families. A 15‐year‐old male patient with a medical history of febrile seizures and a normal delivery experienced his first episodes of right‐sided eye deviations, oropharyngeal twitching, right‐sided upper limb twitching, and salivation at nine. He exhibited limited verbal capability to a few syllables during his seizures. Since then, he has experienced mildly imprecise articulation during the interictal phase. The EEG revealed bilateral centrotemporal spikes with a spike–wave index of 50–60%. MEG revealed left temporal dipoles. These findings led to the diagnosis of SeLECTS in this patient. Valproate provided limited improvements, while the add‐on of Levetiracetam significantly reduced seizure duration from an average of 4–5 min to a few seconds. The follow‐up showed that he is now seizure‐free, and his speech has returned to normal. Another 23‐year‐old female with normal development experienced her first seizure at six. Her seizures occurred exclusively during sleep and were characterized by eye blinking, throat vocalizations, clonic activities of both upper limbs, and a slight impairment of consciousness. The EEG revealed highly bisynchronous centrotemporal spikes and multifocal parietal epileptiform discharges, with a spike–wave index of 80%–90%. MEG showed bilateral precentral gyrus dipoles. Based on the clinical status and EEG findings, a diagnosis of EE‐SWAS was made. The combination of Valproate and Levetiracetam failed to relieve clinical or EEG performances, while a three‐month regimen of oral steroids led to remarkably decreased epileptic activities both clinically and electrically. She has been seizure‐free since the steroids were tapered and other medications were weaned some years later. Additionally, the patient received a diagnosis of attention deficit hyperactivity disorder (ADHD) and a slight memory impairment, both of which have shown limited improvements over time.

### 3.3 Functional alterations of *GRIN2A* variants

We did whole‐cell voltage clamp recordings and immunofluorescence staining on HEK293 cells that were transiently transfected with WT or mutant NMDARs to investigate the functional consequences of M828V and I876T, which haven't been studied elsewhere.

**FIGURE 2:**
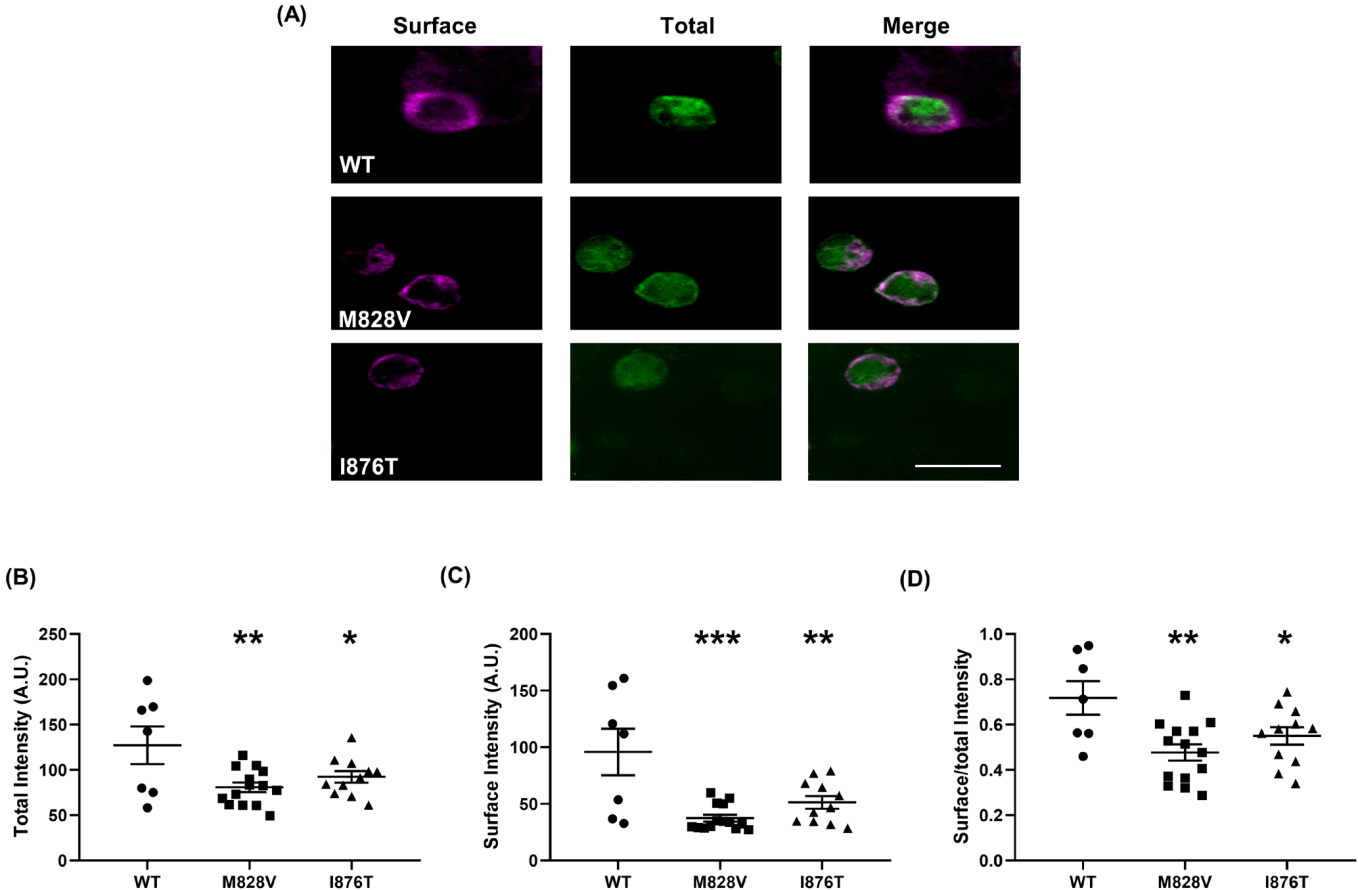
Confocal microscopy of HEK293 cells with co‐expression of WT GluN1 proteins and WT/mutant GluN2A proteins. (A) Surface (magenta) and total (green) expression of GluN1/GluN2A‐WT (top row), GluN1/GluN2A‐M828V (middle row), and GluN1/GluN2A‐I876T (bottom row) detected by immunofluorescence staining in HEK 293 cells. The green signals of GFP represented total expression, while the magenta signals outlined around the transfected HEK 293 cells determined surface expression. Merged images are shown in the right panel. Bar: 20 μm. (B–D) Quantitative analysis of the total and surface expression levels of WT and mutant GluN2A subunits, and their corresponding surface/total expression ratios (WT, *n* = 9; M828V, *n* = 12; I876T, *n* = 10). A.U., Arbitrary Units; One‐way ANOVA with Dunn's multiple comparison test, compared to WT, *p < 0.05, **p < 0.01, ***p < 0.001.

Immunofluorescence staining assessed the expression of mutated subunits (Figure 2A). Cells expressing the M828V and I876T variants demonstrated significantly lower total (*p* < 0.01 for M828V, *p* < 0.05 for I876T, Figure 2B) and surface (*p* < 0.001 for M828V, *p* < 0.01 for I876T, Figure 2C) protein levels to WT cells. To evaluate the relative surface expression, surface/total ratios were assessed, showing decreased membrane delivery of these variants (*p* < 0.01 for M828V, *p* < 0.05 for I876T, Figure 2D).

**FIGURE 3:**
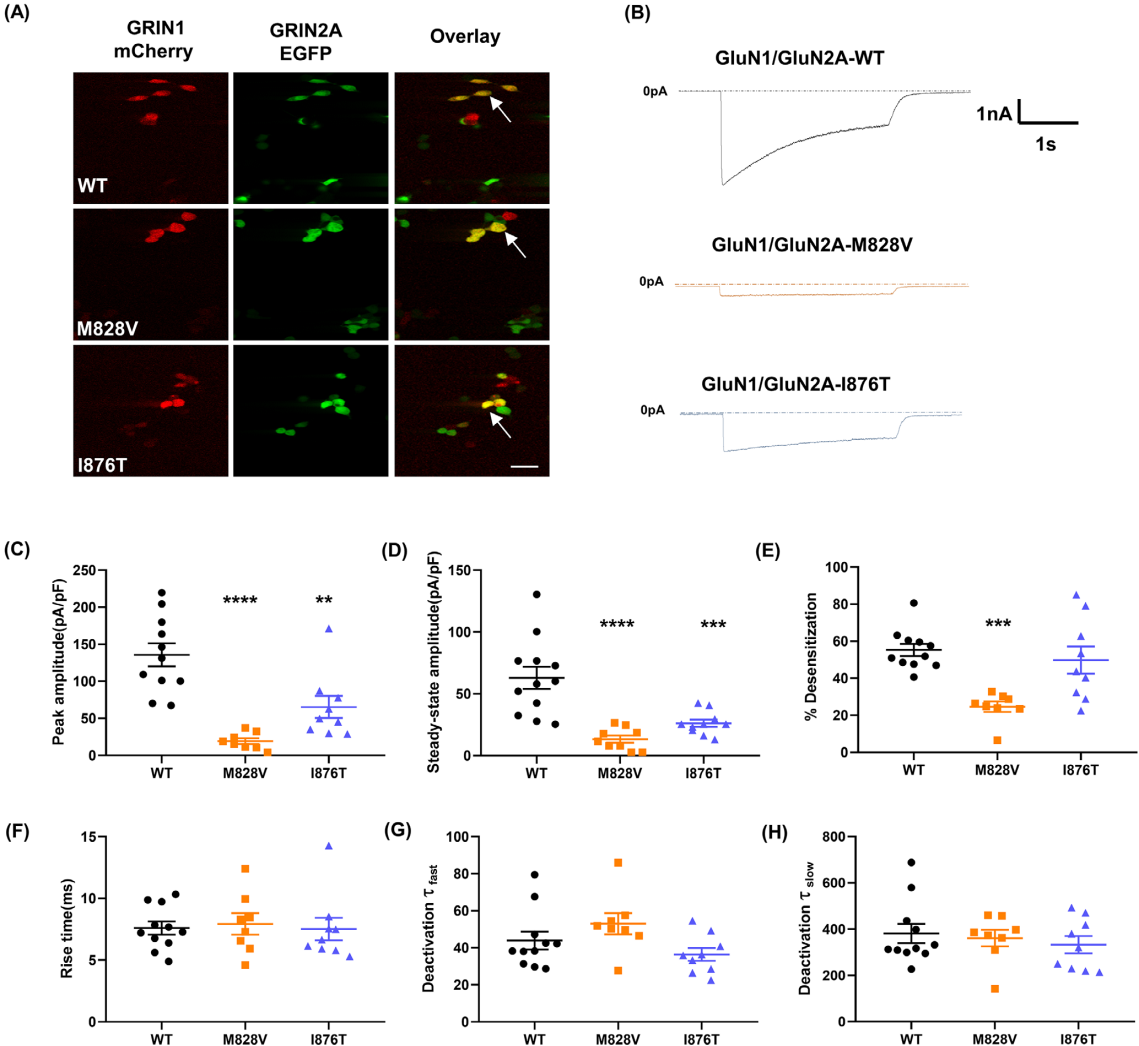
Electrophysiological properties of the WT and mutant GluN1/GluN2A NMDARs. **(A)** HEK293 cells transfected with *GRIN1*‐mCherry and *GRIN2A*‐EGFP genes that were used in whole‐cell recordings. Colocalization appearing in yellow indicates cells with prominent expression of both transfected subunits (white arrows) in the right panel of the overlay view. They were selected for whole‐cell current recordings. Bar: 20 mm. (B) Representative whole‐cell current traces of HEK cells transfected with GluN1/GluN2A‐WT, GluN1/GluN2A‐M828V, or GluN1/GluN2A‐I876T at the holding potential of −70 mV, evoked by 1 mM glutamate and 0.1 mM glycine for 2.5 s (current scale bar, 1 nA; time scale bar, 1 s). (C, D) Peak and steady‐state NMDAR current amplitude in GluN1/GluN2A‐WT or mutant subunit compositions that are normalized to their respective cell sizes (cell capacitance) and quantitatively analyzed (WT, *n* = 12; M828V, *n* = 9; I876T, *n* = 10). One‐way ANOVA with Dunn‘s multiple comparison test, compared to WT, *p < 0.05. (E–H) Summary of channel kinetic properties for the WT and mutant GluN2A subunits co‐expressed with GluN1 subunits, including averaged % Desensitization, rise time, and deactivation time constants (τ _fast_ and τ _slow_) (WT, *n* = 12; M828V, *n* = 9; I876T, *n* = 10). One‐way ANOVA with Dunn's multiple comparison test, compared to WT, **p < 0.01,***p < 0.001,****p < 0.0001.

HEK293 cells that were co‐transfected with both the *GRIN1*‐mCherry and the *GRIN2A*‐EGFP genes were used in whole‐cell recordings (Figure 3A). Representative NMDAR current traces in each group were displayed (Figure 3B), and the current density was calculated (Figure 3C,D). Both the M828V and I876T variant shown dramatically reduced peak (*p* < 0.0001 for M828V, *p* = 0.0019 for I876T) and steady‐state current densities (*p* < 0.0001 for M828V, *p* = 0.0006 for I876T).

Next, we examined whether these variants affect the extent of desensitization (Figure 3E). Variant I876T revealed similar % Desensitization to WT (*p*>0.05). On the other hand, M828V showed a significantly decreased extent of desensitization (*p* < 0.001).

Lastly, some key kinetic parameters for NMDARs were analyzed. As compared with WT, both variants demonstrated similar results in terms of rise time (*p*>0.05 for all, Figure 3F). There were no significant modifications observed in the deactivation time constants for either the fast or slow components in the M828V and I876T variants (*p*>0.05 for all, Figure 3G,H). All detailed statistics for electrophysiological assays can be referred to in Table S3.

### 3.4 Domain‐phenotype‐function correlations of *GRIN2A* variants

**FIGURE 4:**
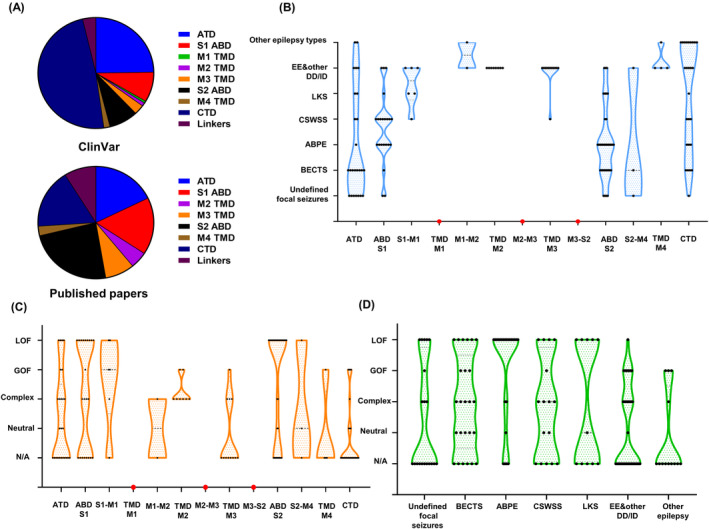
Domain‐phenotype‐function summary of *GRIN2A* variants. (A) Distribution of reported *GRIN2A* variants in the ClinVar database and published papers regarding different protein domains. (B) Summary of epilepsy phenotypes in *GRIN2A* variants from published papers according to different protein domains. Each point indicates an affected individual. (C) Summary of functional consequences of *GRIN2A* variants from published papers with respect to their protein domains. (D**)** Correlations of functional consequences and epilepsy phenotypes in *GRIN2A* variants from published papers. BECTS, benign epilepsy with centrotemporal spikes; ABPE, atypical benign partial epilepsy; CSWSS, continuous spike–wave in slow‐wave sleep; LKS, Landau–Kleffner syndrome; EE, epileptic encephalopathy; ID/DD, intellectual delay/developmental delay; N/A, no data available; GOF, gain‐of‐function; LOF, loss‐of‐function.

First, we categorized *GRIN2A* missense variants sourced from ClinVar by their protein domains (Figure 4A). Nearly half of the total 812 variants were in the CTD region, with the ATD region second. The LBD also contained a considerable number of variants, while occurrences in the TMD and linkers were rare. It should be noted that not all disease‐related variants in ClinVar are pathogenic or causative. Some may be benign or synergistically implicated in the disease. Pathogenic or possibly pathogenic variants are more common in published articles, so we compiled 95 *GRIN2A* missense variants found in 150 affected people from 49 publications (Table S4) to understand the state‐of‐the‐art research and further illustrate the domain‐phenotype‐function correlations. Among them, ATD and LBD variants appeared most frequently. TMD variants were rare in ClinVar but extensively studied in published papers, indicating that few of them are considered benign in this domain. The CTD was relatively understudied despite being occupied with variants.

The spatial distribution of *GRIN2A* variants and their phenotypes was summarized (Figure 4B). ATD and LBD were more commonly associated with the milder range of the disease spectrum, such as BECTS, ABPE, and CSWSS. Severe phenotypes, including EE or DD/ID, were more prevalent in the TMD and linker regions. Apart from EAS, CTD comprised various other epilepsy syndromes, including but not limited to idiopathic generalized epilepsy (IGE, Table S4).

Moreover, the spatial distribution and functional consequences of variants were evaluated (Figure 4C). Sixty‐five of the ninety‐five included variants underwent functional investigations and were categorized. Variants in LBD were mostly LOF, which is plausible due to potentially disrupted agonist binding functions. Variants with complex properties predominated in ATD, TMD, and linkers, indicating that the intricate processes mediated by these regions, such as gating, allosteric regulation, and conformational changes, may pose challenges in determining their functional consequences. Functional data on CTD variants were scarce, revealing GOF effects in patients with IGE or DD.

Regarding the correlation between functional consequences and clinical phenotypes, variants with GOF were more likely to be associated with severe phenotypes and unfavorable prognoses. Notably, patients with EOEE were frequently observed to possess GOF variants.^6^, ^8^ On the other hand, LOF variants demonstrated diverse clinical diagnoses compassing the EAS spectrum (Figure 4D).

## 4 DISCUSSION

*GRIN2A*‐related epilepsy encompasses not only the well‐known epilepsy‐aphasia syndrome but also some developmental and epileptic encephalopathies, even IGE. *GRIN2A* variants are causative in at least 9% of EAS patients, and they occur more frequently in severe phenotypes, ranging from 4.9% in SeLECTS to 17.6% in LKS.^3^, ^4^, ^5^ In Chinese patients with EAS, however, *GRIN2A* was a relatively rare pathogenic gene with a 3.3% detection rate.^16^

This study identified two *GRIN2A* missense variants with high potential for pathogenicity based on in silico functional and structural prediction of damaging effects, which was further confirmed by functional evaluations, providing further insight into the clinical spectrum and mechanisms of *GRIN2A*‐related disorders.

### 4.1 M4‐related variants

The M4 helix of NMDARs is structurally peripheral to the pore‐lining M3 segments and interacts extensively with the phospholipids of CTD.^2^ The M4 helix has fewer neurologically pathogenic missense variants than other domains, and most of them are positioned in the extracellular third of the M4 segment crucial for ion channel gating, with predominant phenotypes involving ID/DD.^8^, ^17^, ^18^, ^19^ Fewer variants were discovered in the bottom two‐thirds of the M4 helix, potentially attributable to the conformationally divergent roles of these two parts. Upon agonist binding, the bottom two‐thirds splay and expand laterally around a conserved Gly residue acting as a pivot. This conformational expansion enables pore opening and maintains the open state of NMDARs, thus facilitating gating and calcium permeation.^20^, ^21^ Based on this pivot point, upper and lower portions of the M4 segment may exhibit different functional impacts, with more severe phenotypes associated with the extracellular end and relatively mild phenotypes linked to those of the bottom portion.

Our study examined the biophysiological and electrophysiological properties of a variant situated within the lower two‐thirds of the M4 segment, M828V. The current density recorded in HEK cells typically represents a combination of the number of functional channels expressed on the surface, the open probability, and the single‐channel conductance. Restricted gating, disrupted receptor biogenesis, or both may be the cause of a significantly reduced current response, which indicates LOF. The M828V variant showed significantly decreased synthesis and surface delivery of receptor protein than the WT, similar to other reported substitutions in the intracellular two‐thirds of the M4 helix.^22^ Conformationally, the residues in the laterally splaying bottom M4 segment have minimal contact with the conducting ions, indicating that alterations in this segment may be less critical for gating activity.^21^ The kinetic parameters of this variant supported the hypothesis, showing limited impacts on deactivation times. Theoretically, the disrupted surface receptor expression is primarily responsible for the reduced current density.

A previously described variant in the upper third M4 segment, M817V, displayed preserved receptor biogenesis, like other variants in the same region. Instead, these subsets of variants typically exhibit significant gating deficits and other channel dysfunctions, potentially leading to the diminished whole‐cell currents.^22^ Our functional analysis further confirmed that the extracellular and intracellular ends of the M4 segment play different roles in channel functions and receptor biogenesis. The way intracellular M4 helix substitutions cause biogenesis deficits warrants further study.

### 4.2 CTD‐related variants

The large, non‐conserved cytoplasmic region was once considered functionally insignificant and pathogenic variant‐free. Recent research has shown that the CTD in NMDARs is essential to multiple physiological functions. It possesses multiple phosphorylation sites for kinases and phosphatases, whose signaling and interactions with intracellular proteins affect receptor expression on the membrane by regulating subcellular localization, trafficking, membrane anchoring, and recycling of NMDAR subunits.^23^, ^24^, ^25^, ^26^

The dysfunctions elicited by CTD variants remain poorly defined, and their causal link with neurological disorders is unclear. Generally, a variant with strong pathogenicity tends to manifest consistent phenotypes, while relatively benign variants have phenotypic discrepancies and mild or paradoxical functional alterations that can be compensated for. For example, the I876T variant was found in a patient with autism spectrum disorder,^27^ and an individual possessing this variant in our study had concurrent EE‐SWAS and ADHD. Functionally, this variant exhibited moderate LOF, with both current density and surface expression decreasing simultaneously. The variety of phenotypes suggests a synergistic, rather than a causative, involvement of the CTD variants in disease development.

### 4.3 Interpretation of complex functional effects

Missense variants could affect one or more biophysiological properties, and their functional consequences can be too complicated to be simply determined as GOF or LOF in practice. It is reasonable to infer that the GOF of NMDARs can be associated with epileptogenesis by generating excessive Ca^2+^ influx into the cell. Despite the fact that glutamatergic receptor hypofunction leading to epilepsy seems paradoxical, there is substantial experimental evidence and hypotheses about the increased excitability of neurons or circuits induced by LOF variants. For instance, *GRIN2A* knockout mice exhibited spontaneous epileptiform discharges and phenotypes similar to EAS.^28^, ^29^ Moreover, NMDAR subunits are found in both glutamatergic neurons and GABAergic interneurons within the human cortex.^30^ The differential impacts of distinct neuron subtypes could perturb the excitatory‐inhibitory balance.^31^, ^32^, ^33^

A complex functional effect can also be difficult to interpret, and the dominant aspect can be perplexing, as in the M828V, where reduced desensitization indicated increased excitability but decreased current density and surface protein expression suggested LOF of the NMDARs. Notably, current density measurements only reflect immediate NMDAR‐mediated activities. It does not capture the broader impact on neuronal and circuit functions. Desensitization, on the other hand, can influence neuronal function and communication within neural networks. To build the panorama of functional outcomes for a given variant, we need as many functional parameters as possible.

There are several limitations in our study. First, an inherent bias in cell selection exists since those cells expressing more NMDARs are prone to die. Hence, relatively healthy cells tend to be present in experiments. Secondly, several key activities, such as posttranslational modifications in the CTD, occur exclusively in a neuronal context. Also, the broader impacts of electrophysiological properties like desensitization may need to be investigated on neuronal or circuit levels instead of non‐excitable cell lines. Therefore, functional investigations of variants in heterologous expression systems may yield limited results. Further experiments in neurons or genetically modified animals, despite being time‐ and resource‐consuming, could provide insights into the overall effects of electrophysiological activities and enhance our understanding of complex variants.

### 4.4 Domain‐phenotype‐function correlations of *GRIN2A* variants

All domains of the GluN2A subunit contain clinically related variants, but pathogenic variants with significant functional alterations cluster with highly conserved domains like LBD, TMD, and linkers, while few benign or likely benign variants are present. CTD does have likely pathogenic variants, despite its scarcity, with a striking diversity of phenotypes. It is worth noting that nearly all *GRIN2A* variants associated with IGE were found in the CTD region.

As for the functional consequences, most domains can demonstrate variants with distinct effects. This indicates that functional consequences cannot be easily classified as binary options, and there is no apparent association between the spatial distributions of the variants and their functional effects. *GRIN2A* variants presenting LOF were associated with more variable phenotypes, and most of them were intermediate. It is reported that LOF or null variants with GluN2A haploinsufficiency can be compensated in some way, leading to considerably less severe clinical phenotypes than GOF variants.^34^, ^35^ Most GOF variants are particularly associated with developmental epileptic encephalopathy.

In summary, this study expanded the clinical and functional spectrum of *GRIN2A* variants. A review of previously reported missense variants demonstrated that sub‐regional, functional consequences, and clinical phenotypes were correlated with each other. We need more extensive and comprehensive functional investigations to properly interpret the ever‐growing disease‐related variants of uncertain significance.

## AUTHOR CONTRIBUTIONS

All authors contributed to the conception and design of this study, and the manuscript preparation. YRD, RM, JQH, and LHG collected clinical information of the probands. LZ and RM performed and analyzed the experiments. LZ, PN and YPW analyzed the data.

## FUNDING INFORMATION

This study was supported by the National Key R&D Program (No.2017YFC0907702, No.2021YFC2501401) and a grant from the National Natural Science Foundation of China (grant number 82371461).

## CONFLICT OF INTEREST STATEMENT

The authors declare no conflicts of interest. The authors confirm that they have read the journal's position on issues involved in ethical publication and affirm that this report is consistent with those guidelines.

## ETHICS STATEMENT

This study was performed in accordance with the ethical standards of the Declaration of Helsinki. Approval was obtained from the Ethics Committee of Xuanwu Hospital Capital Medical University (No.KY20182057‐F‐1). Informed consent was obtained from all individual participants' legal guardians to share clinical information including auxiliary examination data (EEG, MRI, MEG etc.), and provide blood samples for genetic testing.

## Supporting information


Table S1.
Table S2.

Table S3.

Table S4.

## Data Availability

The nucleotide sequences were deposited under accession numbers OR419816‐419818 in the Genbank database. Other anonymized data will be shared upon request with any qualified investigator.
