# Supplementary material for: Clinical phenotype and functional influence of GRIN2A variants in epilepsy‐aphasia syndrome
Source: Epilepsia Open. 2024 Oct 30;9(6):2306–18. doi: 10.1002/epi4.13057 (PMC11633710; doi:10.1002/epi4.13057)
Supplement: Supplementary file 1 — Table S1. Table S2. [file EPI4-9-2306-s001.docx]

**Table S1. Predictions for the pathogenicity of *GRIN2A* variants by the in-silico tools**

|  | **c.2482A>G/****p.Met828Val** | **c.2627T>C/****p.Ile876Thr** |
| --- | --- | --- |
| **SIFT** | 0.004 Deleterious | 0.074 Tolerated |
| **Polyphen-2_HDIV** | 0.995 Possibly Damaging | 0.991 Possibly Damaging |
| **DEOGEN2** | 0.8098 Pathogenic | 0.4881 tolerated |
| **MutationAssesson (functional impact)** | 2.84 medium | 2.765 medium |
| **Mutation Taster** | disease causing | disease causing |
| **PROVEAN** | -3.7 uncertain | -1.69 Neutral |
| **CADD** | 25.2 likely benign | 24.7 likely benign |
| **Alphamissense** | 0.576 likely pathogenic | 0.406 ambiguous |
| **REVEL** | 0.475 uncertain | 0.52 likely disease-causing |
| **MetaRNN** | 0.8361 pathogenic | 0.02636 tolerated |
| **PhastCons100way** | 1.0 | 1.0 |

**Table S2. Minor allele frequency in gnomAD for *GRIN2A* variants identified in this study**

|  | **Allele frequency in all populations** | **Allele frequency in East Asian populations** |
| --- | --- | --- |
| **c.2482A>G/p.Met828Val** | - | - |
| **c.2627T>C/p.Ile876Thr** | 0.000335(exomes)  0.000223(genomes) | 0.003760 |

Abbreviations: gnomAD, Genome Aggregation Database
