# Supplementary material for: Clinical phenotype and functional influence of GRIN2A variants in epilepsy‐aphasia syndrome
Source: Epilepsia Open. 2024 Oct 30;9(6):2306–18. doi: 10.1002/epi4.13057 (PMC11633710; doi:10.1002/epi4.13057)
Supplement: Supplementary file 2 — Table S3. [file EPI4-9-2306-s003.docx]

**Table S3. Summary of electrophysiological data on the WT and variant NMDARs**

|  | **WT(n=12)** | **M828V(n=9)** | **I876T(n=10)** |
| --- | --- | --- | --- |
| **Peak amplitude(pA/pF)** | 135.8±15.6 | 19.3±4.0**** | 65.4±14.9** |
| **Steady-state amplitude(pA/pF)** | 62.9±8.9 | 13.4±8.9**** | 26.2±3.0*** |
| **%Desensitization** | 55.3±3.3 | 24.6±2.8*** | 49.8±7.4 |
| **Rise time(ms)** | 7.6±0.5 | 7.9±0.9 | 7.5±0.9 |
| **Deactivation τ_fast_ (ms)** | 44.0±4.8 | 53.0±5.7 | 36.4±3.5 |
| **Deactivation τ_slow_ (ms)** | 381.5±41.7 | 361.3±35.8 | 333.0±37.1 |
| **Deactivation Amplitude_fast_ (pA)** | 293.2±55.3 | 46.3±8.9*** | 71.6±34.1** |
| **Deactivation Amplitude_slow_ (pA)** | 2169.0±294.4 | 366.7±73.5**** | 512.7±54.0**** |

Values are presented as mean ± SEM. n indicates the number of whole-cell recordings. One-way ANOVA with Dunn’ s multiple comparison test, compared to WT, **p < 0.01, ***p < 0.001, ****p < 0.0001
